# Agreement between preoperative ultrasonographic and histopathologic tumor thickness in eccrine porocarcinoma

**DOI:** 10.1016/j.jdin.2026.09.004

**Published:** 2026-09-22

**Authors:** Tokiyoshi Ikoma, Kazuyasu Fujii, Megumi Aoki, Keisuke Goto, Takahisa Tozawa, Masashi Iwata, Kunihiko Shimada, Jun Morinaga, Shigeto Matsushita

**Affiliations:** a Department of Dermato-Oncology, NHO Kagoshima Medical Center, Kagoshima, Japan; b Department of Dermatology, Kindai University Hospital, Osaka, Japan; c Department of Nephrology, Yamaga Chuo Hospital, Yamaga, Japan

**Keywords:** eccrine porocarcinoma, histopathology, skin cancer, tumor thickness, ultrasonography

*To the Editor:* Eccrine porocarcinoma (EPC) is a rare cutaneous adnexal carcinoma.1^1^ Histopathologic tumor thickness has been associated with adverse outcomes in EPC.2^2^ Correlation and agreement between ultrasonographic and histopathologic tumor thickness have been evaluated in melanoma and basal cell carcinoma,3^3^^,^^4^ whereas published ultrasonographic data on EPC remain limited to isolated reports.1^1^^,^^5^ We therefore evaluated agreement between preoperative ultrasonographic and histopathologic tumor thickness in EPC.

This retrospective study included 35 consecutive patients with histopathologically diagnosed EPC treated at National Hospital Organization Kagoshima Medical Center from October 2014 through May 2025. Archival slides were available for re-review in 32 cases; the 3 cases without available slides were not included in the primary analysis. The primary analysis included 25 cases with paired ultrasonographic and histopathologic thickness measurements. Ultrasonographic images were reviewed by 2 authors, and findings were determined by consensus. On longitudinal scans, ultrasonographic thickness was measured from the epidermal surface to the deepest identifiable hypoechoic extent, including in tumors with ill-defined margins. Histopathologic thickness was measured from the top of the granular layer, or from the ulcer base when present, to the deepest point of invasion. Pearson correlation and linear regression assessed association, and Bland–Altman analysis assessed agreement. Clinical, dermoscopic, ultrasonographic, histopathologic, and outcome characteristics of the full cohort are provided in the Supplementary Material, available via Mendeley at https://data.mendeley.com/datasets/m7jgnczkzj/1.

Ultrasonographic thickness strongly correlated with histopathologic thickness (Pearson r = 0.94; 95% confidence interval, 0.867–0.974; *P* < .001; R^2^ = 0.884) (Fig 1). Bland–Altman analysis showed slight overestimation by ultrasonography, with a mean difference of +0.74 mm and 95% limits of agreement from −1.84 to +3.32 mm (Fig 2). All 25 tumors were hypoechoic, 20 had ill-defined margins, and 22 showed internal Doppler vascular signals. The full cohort had a median age of 83 years, and lesions occurred most commonly on the head and neck and lower extremities. Among the 32 cases with archival slides available for re-review, infiltrative growth was present in 22 (68.8%). During a median follow-up of 23.5 months, 4 patients developed recurrence or metastasis after initial treatment, and 3 died of EPC. These secondary findings are reported descriptively in the Supplementary Material, available via Mendeley at https://data.mendeley.com/datasets/m7jgnczkzj/1and were not used to establish a prognostic classification.

**Fig 1:**
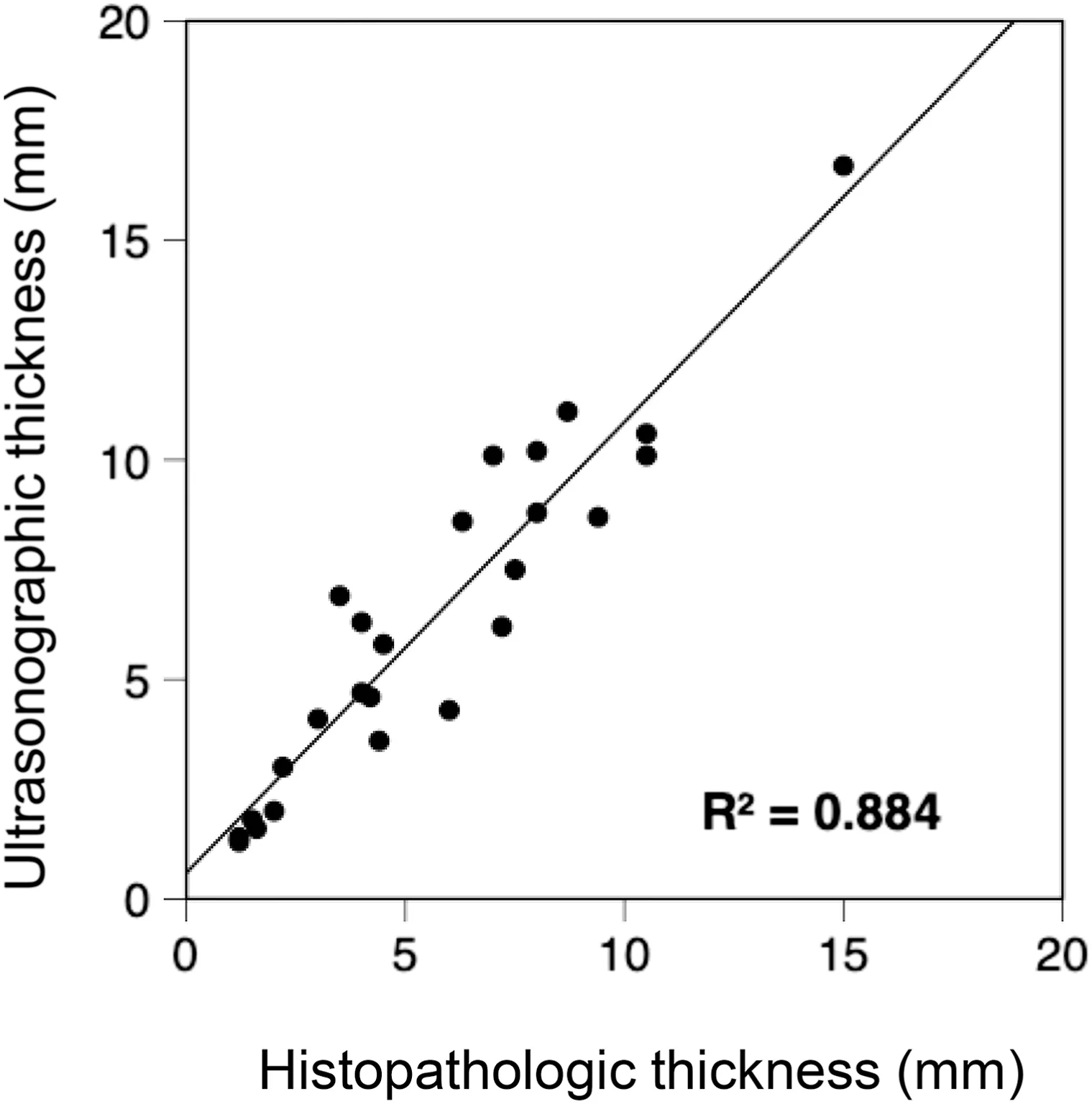
Correlation between preoperative ultrasonographic and histopathologic tumor thickness in eccrine porocarcinoma. Scatter plot of paired measurements in 25 tumors (Pearson r = 0.94; 95% confidence interval, 0.867–0.974; *P* < .001; R^2^ = 0.884). The solid line represents the fitted regression line.

**Fig 2:**
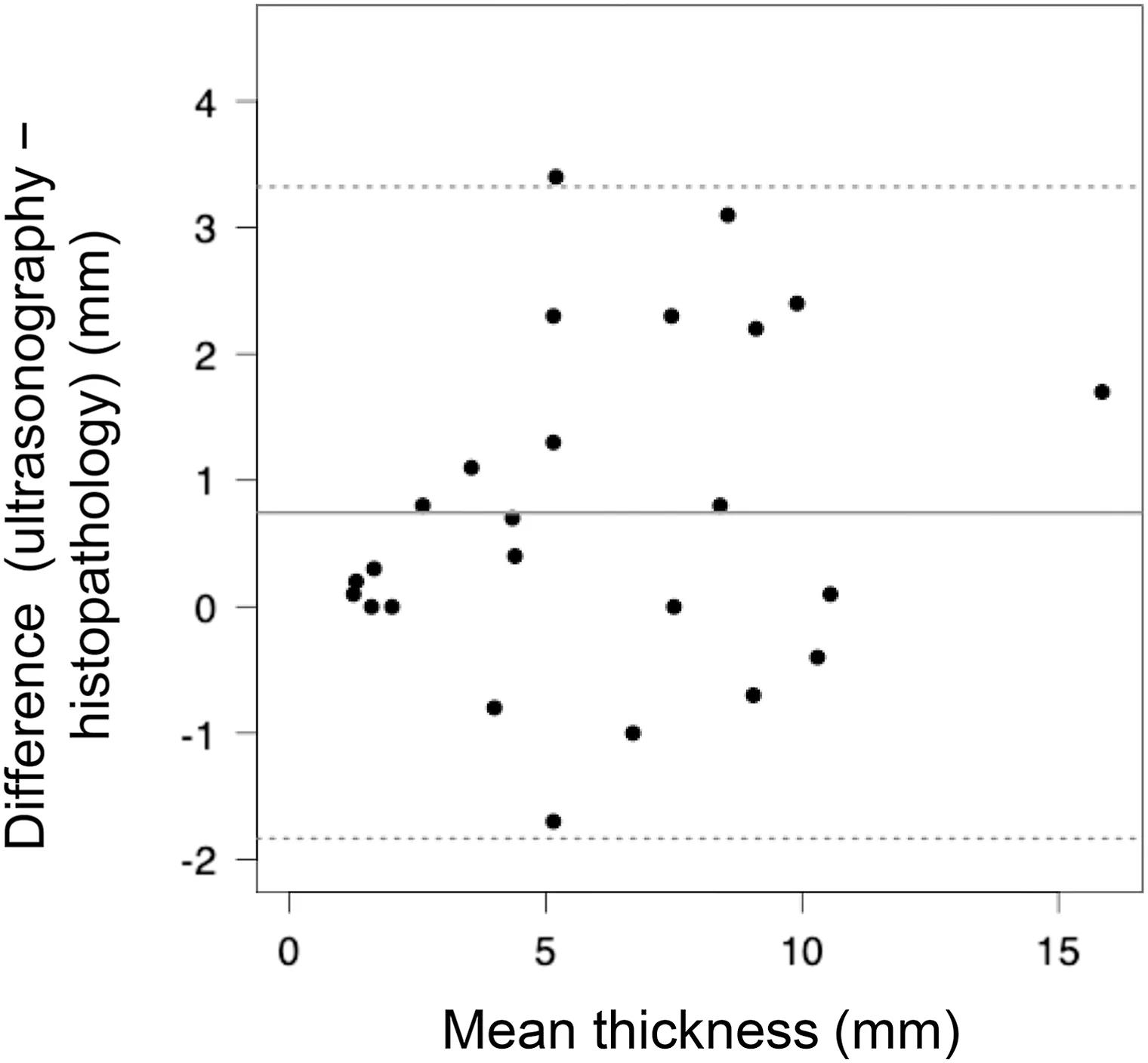
Agreement between preoperative ultrasonographic and histopathologic tumor thickness in eccrine porocarcinoma. Bland–Altman plot of paired measurements in 25 tumors, showing a mean difference (ultrasonography minus histopathology) of +0.74 mm and 95% limits of agreement from −1.84 to +3.32 mm.

Ultrasonography may provide an approximate preoperative estimate of histopathologic tumor thickness in EPC. However, strong correlation does not imply interchangeability at the individual-patient level, as differences of several millimeters occurred. Ultrasonographic and histopathologic measurements were not prospectively matched to the same intratumoral location. The high frequency of ill-defined ultrasonographic margins and use of stored images may also have affected boundary identification. Differences in equipment, operators, and histopathologic sectioning planes may also have contributed. The retrospective single-center design, incomplete imaging availability, and limited outcome events further restrict interpretation. Accordingly, these data do not establish prognostic utility or a clinically actionable thickness threshold and do not justify changes in surgical margins or surveillance. Prospective studies using predefined, spatially matched measurement sites are needed to assess reproducibility and clinical utility.

### Declaration of generative AI and AI-assisted technologies in the writing process

During manuscript preparation, the authors used ChatGPT (OpenAI) to assist with English-language refinement of author-written text. The tool was not used for data collection, data analysis, data interpretation or figure generation. All AI-assisted output was critically reviewed, edited and approved by the authors, who take full responsibility for the final content.

## Conflicts of interest

None disclosed.
